# Characteristics of Professional Self-Concept in Emergency Nurses and Its Association With Burnout and Psychological Resilience

**DOI:** 10.62641/aep.2243

**Published:** 2026-10-15

**Authors:** Limin Hou, Xiao Liang, Xuelian Sun, Fangyan Liu, Binru Han

**Affiliations:** ^1^Department of Emergency, Xuanwu Hospital of Capital Medical University, National Clinical Research Center for Geriatric Diseases, 100053 Beijing, China; ^2^Nursing Department, Xuanwu Hospital of Capital Medical University, 100053 Beijing, China

**Keywords:** emergency nursing, self-concept, burnout, psychological, resilience, psychological

## Abstract

**Background:**

To explore the relationship between professional self-concept and burnout as well as psychological resilience among emergency nurses.

**Methods:**

A cross-sectional questionnaire survey was conducted among 107 emergency nurses at Xuanwu Hospital of Capital Medical University using the Arthur Professional Self-Concept Scale for Nurses, the Chinese Maslach Burnout Inventory (CMBI), and the Chinese Version of the Connor-Davidson Resilience Scale (CD-RISC). Scale scores were compared across demographic characteristics. Pearson correlation analysis was used to examine relationships between professional self-concept, burnout, and psychological resilience, and non-conditional logistic regression was employed to identify influencing factors.

**Results:**

Burnout scores varied significantly by age, work experience, professional title, marital status, child status, and family caregiving responsibilities (all *p* < 0.05). Psychological resilience scores differed significantly by work experience, professional title, child status, and family caregiving responsibilities (all *p* < 0.05). Professional self-concept was significantly negatively correlated with burnout (r = –0.674, *p* = 0.003) and significantly positively correlated with psychological resilience (r = 0.646, *p* = 0.011). Multivariable logistic regression analysis identified older age, longer work experience, senior professional title, and family caregiving responsibilities as significant risk factors for burnout, while higher professional self-concept was a protective factor (all *p* < 0.05). Longer work experience, senior professional title, having children, and higher professional self-concept were associated with greater psychological resilience, whereas family caregiving responsibilities were associated with lower psychological resilience (all *p* < 0.05).

**Conclusions:**

Numerous factors influence burnout and psychological resilience among emergency nurses. A stronger professional self-concept is associated with lower levels of burnout and greater psychological resilience.

## Introduction

The emergency department is characterized by high workload, high-risk exposure, and situational uncertainty [1]. Emergency nurses must maintain constant alertness to manage acute medical events, predisposing them to burnout [2, 3]. Burnout impairs nurses’ mental health, weakens psychological resilience, and compromises patient safety [4, 5]. Mitigating burnout and enhancing resilience among emergency nurses has therefore become a critical priority. Existing research has focused largely on external organizational factors such as staffing and scheduling [6]. While effective to some extent, these approaches are often constrained by systemic resources and difficult to sustain [7]. Internal psychological resources, which are more amenable to individual-level intervention, have received comparatively less attention. Professional self-concept the cognitive evaluation of one’s professional competence and value is a modifiable psychological resource [8]. Studies in general nursing populations have shown its negative association with burnout and positive association with resilience [9, 10, 11]. However, research specifically targeting emergency nurses remains scarce, despite their unique occupational stressors, including high-acuity encounters and frequent trauma exposure [12]. To date, no study has systematically examined the triadic relationship between professional self-concept, burnout, and resilience in emergency nurses. This study aims to address this gap by investigating these associations, providing empirical evidence for developing cognitively oriented interventions tailored to emergency settings.

## Materials and Methods

### General Information

A cross-sectional study was conducted. Data were collected from emergency nurses at Xuanwu Hospital of Capital Medical University between January 2024 and December 2024. Inclusion criteria: (1) Nurse age ≥18 years; (2) Possession of a valid nurse practice license and registration; (3) Voluntary participation in the survey; (4) Engaged in clinical nursing work in the emergency department for ≥1 year. Exclusion criteria: (1) History of major trauma within the past 3 months; (2) Major psychological stress events within the past 3 months; (3) Nurses in training, standardized training, or internship; (4) Nurses on continuous leave due to illness or personal reasons for more than 1 month; (5) Nurses participating in other studies; (6) History of depression, anxiety disorders, or bipolar disorder; diagnosis of a serious medical condition (e.g., cancer, severe infection, metabolic disorders, endocrine disorders, etc.). (7) Incomplete questionnaires, invalid questionnaires, or questionnaires containing logical inconsistencies.

A total of 149 emergency nurses were assessed for eligibility between January 2024 and December 2024. Among them, 15 were excluded due to not meeting the inclusion criteria (nursing experience in the emergency department <1 year). Among the 134 eligible participants, 12 were further excluded due to incomplete questionnaires (missing items >10%), 10 were excluded due to patterned responses (e.g., all items selecting the same answer), and 5 were excluded due to logical errors or inconsistency. Ultimately, 107 valid questionnaires were included in the final analysis.

### Survey Scales

#### General Information Questionnaire

Designed by the researchers based on the study purpose and characteristics of emergency nursing work. It covered demographic characteristics and work background information of the subjects, specifically including: gender, age, education level (college diploma or below, bachelor degree or above), work experience (years), professional title (primary or below, intermediate, senior), employment type (contract-based, permanent staff), marital status (unmarried, married), child status (yes, no), and whether they undertook family caregiving responsibilities (caring for the elderly, patients, or young children).

#### Arthur Nursing Professional Self-Concept Scale

The Arthur Nursing Professional Self-Concept Scale [13] was originally developed by Arthur (1995) and comprises 30 items. The Chinese version of this scale [14] used in the present study consists of five dimensions (leadership, skill, flexibility, satisfaction, and communication), each rated on a 4-point Likert scale. Higher total scores indicate a more positive professional self-concept. The Cronbach’s α coefficient in this study was 0.827.

#### Chinese Maslach Burnout Inventory (CMBI)

The CMBI [15, 16] is a localized instrument based on the three-dimensional burnout framework. It consists of 15 items covering exhaustion, depersonalization, and reduced personal accomplishment. Each item is rated on a 7-point Likert scale, with higher total scores indicating greater burnout. The Cronbach’s α coefficient in this study was 0.841. In accordance with the scoring criteria of the CMBI, burnout was defined as present when the score of any one dimension reached the critical value: exhaustion ≥25, depersonalization ≥11, or reduced personal accomplishment ≥16; zero-burnout (all three dimensions below the critical values) was defined as burnout absent.

#### Chinese Version of the Connor-Davidson Resilience Scale (CD-RISC)

The Chinese version of the CD-RISC [17] consists of 25 items across three dimensions (toughness, strength, and optimism), each rated on a 5-point Likert scale (0–4). The total score ranges from 0 to 100, with higher total scores indicating better psychological resilience. The Cronbach’s α coefficient in this study was 0.894. In the absence of a validated clinical cutoff for this population, psychological resilience level was categorized using the sample median: scores above the median were classified as “strong” resilience, and those at or below the median as “weak” resilience.

### Data Collection and Quality Control

Surveys were conducted on-site by trained research team members. During non-peak work hours, the study purpose and confidentiality principles were explained, and informed consent was obtained from all participants. Questionnaires were completed anonymously and independently, without discussion among colleagues, and were collected on-site upon completion. In the event that participants experienced emotional distress while completing the questionnaire, investigators would immediately pause the survey, provide necessary emotional support, and inquire whether the participant wished to continue or withdraw. Participants were informed that they could discontinue participation at any time without any consequences. If needed, referrals to the hospital’s psychological counseling services were available.

Quality control measures included: uniform training for survey team members prior to data collection; neutral clarification of items during the survey without inducing responses; and immediate completeness checks after collection, with secondary verification to exclude invalid questionnaires (e.g., patterned responses, >10% missing items, or logical errors). Final data were double-entered by two independent researchers, and consistency checks were performed to ensure accuracy.

### Statistical Methods

All analyses were performed using R version 4.0.0 (R Foundation for Statistical Computing, Vienna, Austria). Age was categorized as ≤25, 26–35, and ≥36 years for descriptive comparisons, and work experience was dichotomized at 5 years. Normally distributed continuous variables are presented as mean ± standard deviation (SD). Two-group comparisons were performed using independent-samples *t*-tests, and comparisons among three or more groups were performed using one-way analysis of variance (ANOVA). Categorical variables are presented as frequency (percentage) and were compared using the chi-square test. Pearson correlation analysis was used to examine associations among professional self-concept, burnout, and psychological resilience scores. Multivariable logistic regression models were constructed to identify factors associated with burnout (no = 0, yes = 1) and psychological resilience (weak = 0, strong = 1). Variables with statistical significance in univariate analyses and professional self-concept score were entered into the models. For the burnout model, age, work experience, professional title, family caregiving responsibility, and professional self-concept score were included after excluding marital status and child status because of multicollinearity. For the psychological resilience model, work experience, professional title, child status, family caregiving responsibility, and professional self-concept score were included. Multicollinearity was assessed using the variance inflation factor (VIF), and variables with VIF ≤ 5 were retained. All tests were two-tailed, and *p* < 0.05 was considered statistically significant.

## Results

### Arthur Nursing Professional Self-Concept Scale Scores of the 107 Subjects

Among the 107 subjects, there were 28 aged ≤25 years, 59 aged 26–35 years, and 20 aged ≥36 years; 8 were male nurses and 99 were female nurses. Analysis showed that nurses’ professional self-concept scores were significantly influenced by various demographic characteristics. Regarding age, the ≥36 years age group had the lowest scores, significantly lower than the younger groups. Regarding work experience, nurses with ≤5 years of experience had higher scores than those with longer experience. Regarding professional title, a trend of higher titles associated with lower scores was observed, with primary title holders having the highest scores and senior title holders the lowest. Regarding personal life factors, unmarried nurses had higher scores than married nurses, nurses without children had higher scores than those with children, and nurses without family caregiving responsibilities had higher scores than those with such responsibilities. All these differences were statistically significant (all *p* < 0.05). See Table 1.

**Table 1.** **Arthur Nursing Professional Self-Concept Scale scores of the 107 subjects**.

Items		Number	Professional self-concept score (points)	*t*/F	*p*
Age				F = 9.810	<0.001
	≤25 years	28	73.9 ± 11.0		
	26–35 years	59	72.1 ± 9.8		
	≥36 years	20	65.3 ± 10.5		
Gender				*t* = 0.277	0.782
	Female	99	71.4 ± 13.0		
	Male	8	70.1 ± 8.6		
Education level				*t* = 1.228	0.222
	College or below	84	72.0 ± 11.9		
	Bachelor or above	23	68.7 ± 9.4		
Work experience (years)				*t* = 2.307	0.023
	≤5 years	32	75.1 ± 10.3		
	>5 years	75	69.7 ± 11.4		
Professional title				F = 13.273	<0.001
	Primary or below	49	73.6 ± 10.7		
	Intermediate	52	70.2 ± 12.3		
	Senior	6	62.1 ± 6.2		
Employment type				*t* = –1.295	0.198
	Contract	72	70.4 ± 10.9		
	Permanent staff	35	73.2 ± 9.6		
Marital status				*t* = 3.660	<0.001
	Unmarried	55	75.4 ± 12.2		
	Married	52	67.0 ± 11.5		
Child status				*t* = 2.872	0.005
	No	38	75.8 ± 10.8		
	Yes	69	68.8 ± 12.7		
Family caregiving responsibility				*t* = –4.883	<0.001
	Yes	66	66.9 ± 12.6		
	No	41	78.4 ± 10.5		

Data are presented as mean ± SD. Comparisons between two groups were performed using independent samples *t*-test; comparisons among three or more groups were performed using one-way ANOVA.

### CMBI Scores of the 107 Subjects

Nurses aged ≥36 years had the highest CMBI scores (50.4 ± 9.7), followed by those aged 26–35 years (44.6 ± 7.3), and those aged ≤25 years had the lowest scores (43.1 ± 8.9). Nurses with >5 years of work experience had significantly higher CMBI scores (47.3 ± 9.7) than those with ≤5 years (40.7 ± 8.0). Nurses with senior professional titles had the highest CMBI scores (50.8 ± 6.5), followed by those with intermediate titles (45.8 ± 8.4), and those with primary or lower titles had the lowest scores (44.1 ± 8.3). Married nurses had significantly higher CMBI scores (48.5 ± 8.5) than unmarried nurses (42.3 ± 8.9). Nurses with children had higher CMBI scores (47.2 ± 9.4) than those without children (41.9 ± 8.1). Nurses with family caregiving responsibilities had higher CMBI scores (48.8 ± 9.6) than those without such responsibilities (39.7 ± 8.8). All differences were statistically significant (all *p* < 0.05). See Table 2.

**Table 2.** **CMBI scores of the 107 subjects**.

Items		Number	CMBI score (points)	*t*/F	*p*
Age				F = 9.102	<0.001
	≤25 years	28	43.1 ± 8.9		
	26–35 years	59	44.6 ± 7.3		
	≥36 years	20	50.4 ± 9.7		
Gender				*t* = –0.728	0.468
	Female	99	45.1 ± 10.3		
	Male	8	47.8 ± 6.5		
Education level				*t* = 0.862	0.390
	College or below	84	45.7 ± 9.6		
	Bachelor or above	23	43.8 ± 8.4		
Work experience (years)				*t* = –3.386	0.001
	≤5 years	32	40.7 ± 8.0		
	>5 years	75	47.3 ± 9.7		
Professional title				F = 13.203	<0.001
	Primary or below	49	44.1 ± 8.3		
	Intermediate	52	45.8 ± 8.4		
	Senior	6	50.8 ± 6.5		
Employment type				*t* = –1.755	0.082
	Contract	72	44.2 ± 9.5		
	Permanent staff	35	47.6 ± 9.2		
Marital status				*t* = –3.681	<0.001
	Unmarried	55	42.3 ± 8.9		
	Married	52	48.5 ± 8.5		
Child status				*t* = –2.927	0.004
	No	38	41.9 ± 8.1		
	Yes	69	47.2 ± 9.4		
Family caregiving responsibility				*t* = 4.919	<0.001
	Yes	66	48.8 ± 9.6		
	No	41	39.7 ± 8.8		

Data are presented as mean ± SD. CMBI, Chinese Maslach Burnout Inventory. Comparisons between two groups were performed using independent samples t-test; comparisons among three or more groups were performed using one-way ANOVA.

### CD-RISC Scale Scores of the 107 Subjects

Nurses with ≤5 years of work experience had significantly lower psychological resilience scores (51.8 ± 9.8) than those with >5 years of experience (58.2 ± 12.6). Psychological resilience scores increased with higher professional titles, being lowest for primary or lower titles (54.4 ± 9.8), intermediate for intermediate titles (57.1 ± 12.3), and highest for senior titles (64.9 ± 8.8). Nurses without children had lower resilience scores (52.3 ± 10.5) than those with children (58.2 ± 12.8). Nurses without family caregiving responsibilities had significantly higher resilience scores (62.6 ± 11.7) than those with responsibilities (52.4 ± 12.5). All differences were statistically significant (all *p* < 0.05). See Table 3.

**Table 3.** **CD-RISC scale scores of the 107 subjects**.

Items		Number	CD-RISC score (points)	*t*/F	*p*
Age				F = 2.291	0.227
	≤25 years	28	55.1 ± 8.6		
	26–35 years	59	56.5 ± 12.5		
	≥36 years	20	57.4 ± 8.3		
Gender				*t* = –0.557	0.579
	Female	99	56.1 ± 13.4		
	Male	8	58.8 ± 9.7		
Education level				*t* = –0.952	0.343
	College or below	84	55.7 ± 12.8		
	Bachelor or above	23	58.5 ± 11.3		
Work experience (years)				*t* = –2.559	0.012
	≤5 years	32	51.8 ± 9.8		
	>5 years	75	58.2 ± 12.6		
Professional title				F = 13.273	<0.001
	Primary or below	49	54.4 ± 9.8		
	Intermediate	52	57.1 ± 12.3		
	Senior	6	64.9 ± 8.8		
Employment type				*t* = –1.525	0.130
	Contract	72	55.1 ± 12.5		
	Permanent staff	35	58.8 ± 10.1		
Marital status				*t* = –1.833	0.070
	Unmarried	55	54.1 ± 12.4		
	Married	52	58.6 ± 13.0		
Child status				*t* = 2.426	0.017
	No	38	52.3 ± 10.5		
	Yes	69	58.2 ± 12.8		
Family caregiving responsibility				*t* = –4.204	<0.001
	Yes	66	52.4 ± 12.5		
	No	41	62.6 ± 11.7		

Data are presented as mean ± SD. CD-RISC, Connor-Davidson Resilience Scale. Comparisons between two groups were performed using independent samples *t*-test; comparisons among three or more groups were performed using one-way ANOVA.

### Correlation Between Professional Self-Concept Scores and Burnout Scores, Psychological Resilience Scores

Linear correlation analysis was conducted on the professional self-concept scores, burnout scores, and psychological resilience scores of the 107 subjects. The results showed that professional self-concept scores were negatively correlated with CMBI scores (r = –0.674, *p* = 0.003) and positively correlated with psychological resilience scores (r = 0.646, *p* = 0.011). See Fig. 1.

**Fig. 1.**
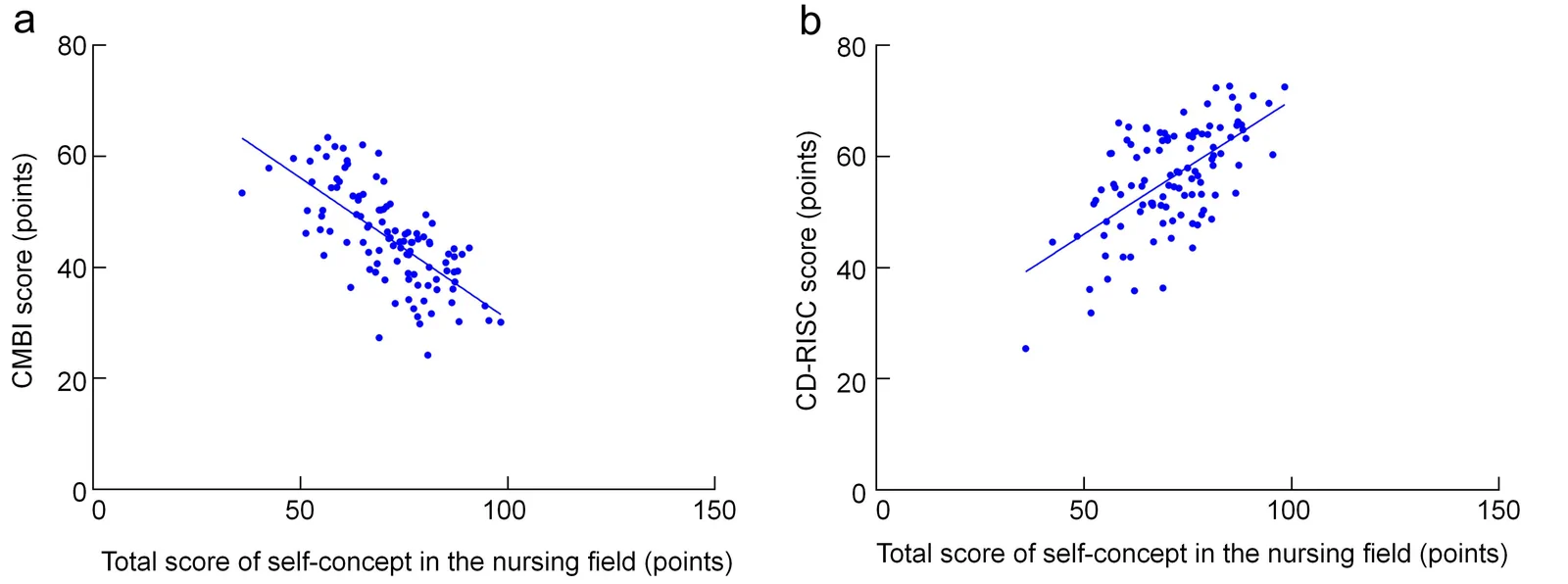
**Scatter plots of the correlation between professional self-concept scores and burnout scores (a), as well as between professional self-concept scores and psychological resilience scores (b)**. CMBI, Chinese Maslach Burnout Inventory; CD-RISC, Connor-Davidson Resilience Scale.

### Analysis of Influencing Factors of Burnout in Emergency Nurses

Using the statistically significant single factors from Table 2 as independent variables and the presence or absence of burnout in emergency nurses (no = 0, yes = 1) as the dependent variable, logistic regression model analysis was conducted. After analysis using the VIF method and excluding the two independent variables of marital status and child status, the logistic regression model results showed: older age, longer work experience, having a senior professional title, and having family caregiving responsibilities were risk factors for burnout in emergency nurses (*p* < 0.05), while a stronger professional self-concept could reduce the probability of burnout in emergency nurses (*p* < 0.05). See Table 4.

**Table 4.** **Analysis of influencing factors of burnout in emergency nurses**.

Indicator		β	SE	Wald	*p*	OR	95%CI
Age		0.017	0.007	5.898	0.005	1.017	1.003~1.031
Work experience		1.982	0.833	5.661	0.013	7.257	1.418~37.139
Professional title							
	Primary or below					1.000	
	Intermediate	0.101	0.081	1.555	0.342	1.106	0.944~1.297
	Senior	1.302	0.557	5.464	0.018	3.677	1.234~10.954
Family caregiving responsibility							
	No					1.000	
	Yes	1.208	0.511	5.588	0.014	3.347	1.229~9.112
Professional self-concept score		–0.107	0.036	8.834	0.024	0.899	0.837 ~0.964
Constant		1.309	0.660	3.934	0.057		

SE, standard error; OR, odds ratio; CI, confidence interval. The reference category for professional title was “primary or below” (OR = 1.000). Variables with VIF > 5 (marital status and child status) were excluded from the final model due to multicollinearity. Burnout present was defined as scoring at or above the critical value on any one or more of the three CMBI dimensions (exhaustion ≥25, depersonalization ≥11, reduced personal accomplishment ≥16) [16].

### Analysis of Influencing Factors of Psychological Resilience in Emergency Nurses

Using the statistically significant variables in Table 3 and the professional self-concept score as independent variables, and psychological resilience level in emergency nurses (weak = 0, strong = 1) as the dependent variable, a logistic regression model was constructed. The results showed that longer work experience, senior professional title, having children, and a higher professional self-concept score were associated with stronger psychological resilience (*p* < 0.05), whereas family caregiving responsibilities were associated with weaker psychological resilience (*p* < 0.05). See Table 5.

**Table 5.** **Analysis of influencing factors of psychological resilience in emergency nurses**.

Indicator		β	SE	Wald	*p*	OR	95%CI
Work experience		0.817	0.355	5.296	0.024	2.264	1.129~4.539
Professional title							
	Primary or below					1.000	
	Intermediate	0.332	0.182	3.328	0.089	1.394	0.976~1.991
	Senior	0.619	0.264	5.498	0.018	1.857	1.107~3.116
Child status							
	No					1.000	
	Yes	1.004	0.405	6.146	<0.001	2.729	1.234~6.036
Family caregiving responsibility							
	No					1.000	
	Yes	–0.694	0.336	4.266	0.048	0.500	0.259~0.965
Professional self-concept score		0.211	0.084	6.310	<0.001	1.235	1.047~1.456
Constant		1.404	0.678	4.288	0.048		

SE, standard error; OR, odds ratio; CI, confidence interval. Strong resilience was defined as CD-RISC total score > sample median; weak resilience was defined as CD-RISC total score ≤ sample median. Reference categories were set as follows: for professional title, “primary or below”; for child status, “No”; for family caregiving responsibility, “No”.

## Discussion

### Emergency Nurses’ Professional Self-Concept is Influenced by Age, Professional Title, and Family Factors

This study showed that nurses’ Arthur Nursing Professional Self-Concept scores were significantly influenced by age, professional title, and family factors. Notably, nurses aged ≥36 years, with more seniority, and with senior professional titles had lower professional self-concept scores compared to younger, lower-titled nurses. This finding differs from the study by Salameh *et al*. [18], possibly due to the specific work environment of the emergency department. As work experience increases, senior nurses accumulate rich clinical experience but are also long-term frontline workers in high-risk, high-load emergency settings, prone to occupational fatigue. Combined with career promotion bottlenecks or confusion about managerial roles, this may lead to a decreased self-evaluation of their professional value [19]. Furthermore, data from this cross-sectional sample indicated that nurses without family caregiving responsibilities, without children, and unmarried had higher professional self-concept scores. This suggests that family burden may be an important factor weakening professional focus. Younger nurses, with fewer family ties, may be able to invest more energy in career exploration and skill enhancement, thereby maintaining a more positive professional self-concept [20]. Professional self-concept can serve as a core cognitive reference for an individual’s assessment of the match between job demands and self-competence. When nurses form a stable positive evaluation of their professional abilities, value, and role competence, they are more inclined to define emergency stress events as controllable professional challenges, thereby adopting problem-focused coping styles and proactive resource mobilization, reducing the risk of emotional exhaustion and depersonalization. When professional self-concept is low, individuals are more likely to define similar events as threatening burdens, potentially leading to avoidance or passive coping, thereby accelerating psychological resource consumption and promoting burnout formation.

### Age, Professional Title, and Family Caregiving Responsibilities are Major Risk Factors for Burnout in Emergency Nurses

The survey results indicated that older age, longer work experience, senior professional title, and undertaking family caregiving responsibilities were risk factors for burnout among nurses (Although being married and having children were associated with higher burnout scores in the univariate analysis, they were excluded from the final multivariable model due to multicollinearity). In this sample, nurses aged ≥36 years and those with senior titles had higher CMBI scores, possibly related to overlapping role loads. This group is responsible for both treating difficult and critically ill patients and undertaking teaching and quality management duties. Role overload can trigger emotional exhaustion [21]. When clinical skills and management experience reach a relatively stable stage, but career advancement channels, specialty development, or scope of authority lack matching improvement, individuals may easily develop a sense of career stagnation and a gap in perceived value return, thereby weakening self-assessment of professional competence and sense of work meaning. Additionally, senior nurses often find themselves in a blurred boundary between the roles of clinical expert and potential manager, requiring frequent switching between treatment efficiency, patient communication, team coordination, and policy implementation. Ongoing role tension can further erode stable professional self-evaluation and accumulate burnout risk. Vaisi-Raygani *et al*. [22] also noted in their review on emergency nurses that the unique shift system, exposure to traumatic events, and lack of management support in the emergency environment are key triggers for burnout. Moreover, nurses with family caregiving responsibilities had significantly higher burnout levels, which is related to the dual pressures faced by the “sandwich generation”. The emergency shift work system makes it difficult to balance work and family. When the family support system is insufficient, physical and mental exhaustion caused by role conflict is projected into work, manifesting as higher levels of burnout [23].

### Psychological Resilience of Emergency Nurses Increases With Seniority but is Constrained by Family Caregiving Responsibilities

This study also found that although senior and high-titled nurses had stronger feelings of burnout, their psychological resilience scores were significantly higher than those of junior nurses. This suggests that psychological resilience reflects more on coping and recovery abilities and is not equivalent to having abundant psychological resources. This is consistent with the results of the meta-analysis by Castillo-González *et al*. [24]. The continuous high-intensity tasks and emotional labor in the emergency department lead to cumulative resource depletion, manifested as increased burnout. On the other hand, repeatedly handling critical situations prompts individuals to form stable coping scripts, risk expectations, and emotion regulation strategies, resulting in higher scores on recovery-related aspects of event-handling capability. Therefore, high resilience can coexist with high burnout, its essence being the parallel enhancement of coping ability and accelerated resource consumption. For nursing management, it is necessary to distinguish between “being able to withstand pressure” and “having a low cost of stress”, avoiding the use of high resilience as a substitute indicator for low burnout [25]. Furthermore, nurses with children had higher psychological resilience, which may be related to parenting experiences enhancing individual responsibility and emotional maturity. However, if the family caregiving burden is too heavy (e.g., caring for the elderly or patients), it can deplete individual psychological resources, leading to a significant decrease in psychological resilience [26, 27]. This reminds nursing managers that when focusing on nurses’ mental health, they should distinguish that strong stress tolerance and mental health are not equivalent concepts; a highly resilient nurse may be in a state of high exhaustion.

### Professional Self-Concept is a Key Protective Factor in Alleviating Burnout and Enhancing Psychological Resilience

Correlation analysis and multivariable logistic regression models showed that professional self-concept was significantly negatively correlated with burnout, and a strong professional self-concept could reduce the incidence of burnout. This is consistent with the conclusions of studies by Goliroshan *et al*. [28] and Liu *et al*. [29]. These scholars confirmed that professional identity can significantly negatively predict burnout and play a mediating role between leadership, perceived competence, and burnout. Professional self-concept is an individual’s self-assessment of professional knowledge, value, and skills, serving as an internal resource for nurses to cope with occupational stress. When nurses have a high level of identification with their professional value and confidence in their professional skills, they can engage in more positive self-attribution and emotion regulation, thereby reducing depersonalization and reduced personal accomplishment caused by work stress [30, 31]. Conversely, nurses with a negative self-concept are more likely to view work as a burden, lack intrinsic achievement motivation, and thus exacerbate burnout [32]. The study by Ren *et al*. [33] further pointed out that psychological resilience plays a partial mediating role between professional identity and burnout, meaning that a positive professional self-perception can buffer burnout by enhancing psychological resilience. According to social cognitive theory, self-concept is a mediating variable for individuals coping with social life events. Emergency nurses with a positive professional self-concept, when facing frustrating situations such as failed rescues or doctor-patient disputes, are more inclined to view them as professional challenges rather than threats. This positive cognitive appraisal helps activate internal potential and mobilize psychological defense mechanisms, thereby demonstrating higher psychological resilience [34].

Furthermore, the multifactorial logistic regression model in this study confirmed that increasing age, senior professional title, and undertaking family caregiving responsibilities were independent risk factors for burnout in emergency nurses. This may stem from the high-load nature of emergency nursing work. Although senior, high-titled nurses have rich clinical experience, they simultaneously bear the heavy responsibilities of handling difficult and critical rescues and management teaching while their physical function gradually declines with age. The contradiction between high responsibility and declining physical strength makes them more susceptible to emotional exhaustion. Family caregiving responsibilities, as an external stressor that primarily depletes personal energy, prevent nurses from obtaining effective rest after intense work, forcing them to continuously face dual role conflicts between work and family, leading to the depletion of psychological resources and significantly increasing the probability of burnout.

### Implementing Tiered Interventions, Strengthening Family Support, and Providing Occupational Cognitive Education are Effective Strategies to Alleviate Burnout in Emergency Nurses

Given the characteristics of high burnout, high resilience, and low self-concept among senior nurses, and the significant impact of family burden on nurses’ psychology, the following strategies are recommended for clinical nursing management: (1) For senior nurses in a career plateau period, clinical mentor-specialty leadership programs can be established. Define their duty boundaries and authority scope in teaching supervision, quality improvement, and complex case review through institutional forms, transforming experiential advantages into transmittable knowledge capital, thereby enhancing professional self-concept in dimensions like leadership and satisfaction. (2) For those with family caregiving responsibilities and higher burnout risk, schedule strategies can be configured at the shift level, including continuous rest periods and predictable shift patterns, and incorporate a department-level substitute nurse pool to reduce peak load from work-family role conflicts. (3) In continuing education, incorporate the enhancement of professional self-concept into practical cognitive training content. Examples include cognitive debriefing based on typical emergency events, construction of competency inventories, and peer supervision feedback mechanisms, encouraging individuals to develop a stable sense of competence and value, thereby enhancing their buffering capacity against burnout.

### Limitations

Several limitations of this study should be acknowledged. First, this was a single-center, cross-sectional study with a relatively small sample size (n = 107) drawn from the emergency department of one hospital, which may limit the generalizability of the findings to other regions, hospital types, or healthcare systems. Second, the cross-sectional design precludes any causal inferences; although significant correlations were observed between professional self-concept, burnout, and psychological resilience, the possibility of bidirectional or reciprocal relationships among these variables cannot be ruled out. Third, the data relied entirely on self-reported questionnaires. Despite the use of anonymous administration and rigorous quality control measures, recall bias and social desirability bias may have influenced the accuracy of the responses. Fourth, this study did not examine organizational-level factors (e.g., management style, team support climate, perceived pay equity) or nurses’ non-work-related circumstances, which may also moderate or mediate the relationships between professional self-concept, burnout, and psychological resilience. Fifth, the descriptive analyses were stratified solely by demographic characteristics rather than by the core study variables (i.e., burnout and psychological resilience levels). Future analyses of this dataset would benefit from stratifying professional self-concept scores directly by burnout and resilience levels to more clearly reveal differences aligned with the study’s primary objective. Future research should employ multicenter, longitudinal designs combined with qualitative approaches to more comprehensively elucidate the dynamic mechanisms linking emergency nurses’ professional cognition and mental health outcomes.

## Conclusions

The investigation revealed that numerous factors influence burnout and psychological resilience among emergency nurses. Among them, a stronger professional self-concept among nursing staff is associated with weaker feelings of burnout and stronger psychological resilience.

## Availability of Data and Materials

The datasets used and/or analyzed during the current study are available from the corresponding author on reasonable request.
